# Prediction of acute multiple sclerosis relapses by transcription levels of peripheral blood cells

**DOI:** 10.1186/1755-8794-2-46

**Published:** 2009-07-22

**Authors:** Michael Gurevich, Tamir Tuller, Udi Rubinstein, Rotem Or-Bach, Anat Achiron

**Affiliations:** 1 Multiple Sclerosis Center, Sheba Medical Center, Tel-Hashomer, Sackler School of Medicine, Tel-Aviv University, Tel Aviv, Israel; 2 School of Computer Science, Tel Aviv University, Tel Aviv, Israel

## Abstract

**Background:**

The ability to predict the spatial frequency of relapses in multiple sclerosis (MS) would enable physicians to decide when to intervene more aggressively and to plan clinical trials more accurately.

**Methods:**

In the current study our objective was to determine if subsets of genes can predict the time to the next acute relapse in patients with MS. Data-mining and predictive modeling tools were utilized to analyze a gene-expression dataset of 94 non-treated patients; 62 patients with definite MS and 32 patients with clinically isolated syndrome (CIS). The dataset included the expression levels of 10,594 genes and annotated sequences corresponding to 22,215 gene-transcripts that appear in the microarray.

**Results:**

We designed a two stage predictor. The first stage predictor was based on the expression level of 10 genes, and predicted the time to next relapse with a resolution of 500 days (error rate 0.079, p < 0.001). If the predicted relapse was to occur in less than 500 days, a second stage predictor based on an additional different set of 9 genes was used to give a more accurate estimation of the time till the next relapse (in resolution of 50 days). The error rate of the second stage predictor was 2.3 fold lower than the error rate of random predictions (error rate = 0.35, p < 0.001). The predictors were further evaluated and found effective both for untreated MS patients and for MS patients that subsequently received immunomodulatory treatments after the initial testing (the error rate of the first level predictor was < 0.18 with p < 0.001 for all the patient groups).

**Conclusion:**

We conclude that gene expression analysis is a valuable tool that can be used in clinical practice to predict future MS disease activity. Similar approach can be also useful for dealing with other autoimmune diseases that characterized by relapsing-remitting nature.

## Background

Multiple sclerosis (MS) is an autoimmune demyelinating central nervous system (CNS) disease characterized by an unpredictable relapsing-remitting course. In MS and other autoimmune diseases, a relapse is defined as the new onset or worsening of clinical neurological symptoms, and is followed by periods of remissions with no disease activity. Relapses are the basic feature of MS and other autoimmune diseases such as myasthenia gravis [1], systemic lupus erythemathosus [2], rheumatoid arthritis [3], and Crohn's disease [4]. In MS, relapses are the consequence of complex immunological and neurodegenerative processes. Relapses in MS are associated with myelin and axonal loss; they may cause new acute inflammatory lesions or can activate old lesions within the CNS [5-7]. Accordingly, relapses are the visible clinical expression of the complicated immunopathological mechanisms operating in the CNS and peripheral blood. The ability to predict the occurrence of a subsequent relapse (yes/no) and to estimate the time when that process will occur has important clinical and practical implications. This knowledge can help in decisions related to treatment – *e.g. *either treat patients with more aggressive disease or avoid over-treatment of patients with a more favorable disease course. Prediction of the time to next relapse can also be useful in the design of clinical trials as an additional criterion for selecting active patients. For patients with clinically isolated syndrome (CIS), who have just experienced the first relapse, such a tool can be used for estimating the probability to convert to definite MS by predicting the time until the second relapse.

Biologically, analysis of genes and pathways that are related to predicting relapses may help to better understand the mechanisms underlying the progression of the disease, and more specifically the processes that trigger and operate in acute MS relapses.

Various demographic and disease-related variables have been utilized for predicting clinical outcome. Late age at disease onset, poly-symptomatic symptomatology at onset, higher annual relapse rate and short time-interval between attacks are correlated with poor outcome, while onset with the presentation of optic neuritis or sensory symptomatology have been associated with a good outcome [8-15]. Disease-related variables, measurements of autoantibodies, and gene expression were found to be useful for diagnosis and prognosis in MS and in other autoimmune diseases [16-18]. For example, in the case of MS, Martinez-Yelamos *et al *[19,20] showed that CSF-TAU and 14-3 3 proteins are independent predictive factors for short time conversion to clinical definite MS. On the other hand, the correlation between anti-myelin antibodies and time to next relapse in CIS patients has produced controversial results [21-23].

Imaging is considered as a more sensitive tool for predicting MS progression; it was reported that various parameters like brain MRI lesion load including the number, volume and location of lesions, as well as the presence of enhancing lesions and brain atrophy can predict disease outcome [24]. In CIS patients, T2 lesion volume at onset correlates with disability over the next 10 years, and with the time to progress to definite MS [25].

The possibility to use peripheral blood gene expression analysis for prediction of clinical outcome in MS patients was demonstrated in our previous work [26] where we showed that peripheral blood mononuclear cells (PBMC) gene expression based classifier correctly predicted disease progression for two years. Another relevant work is the work of Baranzini *et al. *[27], they showed that PBMC gene expression can be used to predict the response of MS patients to recombinant human interferon beta (*rINFβ*).

The aim of current study was to evaluate whether it is possible to use peripheral blood gene expression to predict the time to next acute relapse in CIS and relapsing-remitting MS patients.

Most of the new radiological MRI lesions are clinically silent. The frequency of new radiological lesions is ten times higher than the frequency of clinical relapses; i.e., on average, a cumulative effect of about 10 new radiological MRI lesions is equivalent to one clinical relapse [28]. Since most clinical relapses are associated with new MRI lesions, and since MRI measurements was available only for small fraction of the patients, in the current study we focused only on clinically definite MS relapses.

We designed a comprehensive feature selection procedure that was implemented on different sets of feature including: 1) all genes represented in the microarray; 2) set of genes significantly discriminated between groups of different classes of time to next relapse; 3) genes significantly correlated with time to next relapse, and 4) clinical and demographical confounders.

This approach enabled us to identify a PBMC gene expression based predictors that envisioned the time until the next relapse with high accuracy.

## Methods

### Subjects

The study was approved by the Sheba Medical Center Institutional Review Board, and all patients gave written informed consent for participation.

It was a prospective collection of data. The data set includes 94 patients, 62 patients diagnosed with definite MS according to McDonald criteria [29] and 32 patients with CIS. All patients were free of steroids and immunomodulatory treatments for at least 30 days before blood withdrawal, and were at least one year after treatment with cyclophosphamide (see Additional file 1). As can be seen, 62 patients had not experienced previous treatment; the rest of the patients had average distance of 300.4 ± 68.3 days from previous immunomodulatory treatment. The time to next relapse was not a selection criterion for inclusion patients in study; we randomly sampled the blood of 100 patients (94 microarrays passed quality control criteria).

We excluded from the study patients with Neuromylitis Optica (NMO) according to the criteria of Wingerchuk *et al. *[30].

The demographical characteristics of the patients are presented in Table 1. Patients were followed-up prospectively for a maximal period of 3.5 years (1264 days) or up to the first next acute relapse during the follow up period. Neurological examination was performed every 3 months and at the time of a suspected relapse, Expanded Disability Status Scale (EDSS) assessment was completed accordingly.

**Table 1:** Clinical and demographical characteristic of the analyzed patients.

	**Age (Years)**	**Disease Duration (Years)**	**Annual Relapse Rate**	**EDSS**	**F/M**	**Yes/No Future IMD Treatment**
**CIS**	32.1 ± 1.5	0.20 ± 0.02	------	0.9 ± 0.2	19/13	0/32

**Definite MS**	38.5 ± 1.4	8.50 ± 1.09	0.92 ± 0.1	2.4 ± 0.2	41/21	33/29

During the follow-up period, 33 definite MS patients initiated various immunomodulatory treatments (Table 2) while 61 patients remained untreated.

**Table 2:** The immunomodulatory treatments the analyzed patients underwent after blood sampling.

	**CIS**	**Definite MS**	**Non treated patients**	**Interferon β-1a Avonex**	**Interferon β-1b Betaferon**	**Interferon β-1a Rebif**	**Glatiramer acetate Copaxone**	**Intravenous Immunoglobulins Iv-Ig**
**No of Patients**	32	62	61	5	2	10	10	6

As the aim of this study is the prediction of the time till the next relapse we gathered patients with large range of this parameter; our dataset included patients with long period between relapses. Some of these patients have benign MS (if they have EDSS < 3.0 after 15 years with the disease). Patients with benign MS are not necessarily treated in our country. Additionally, CIS patients (32 of the patients) are not treated according medical regulations in our country. Thus, in summery, our study included relatively high number untreated patients

### Definition of Relapses

MS relapse was defined as the onset of new objective neurological symptoms/signs or worsening of existing neurological disability, not accompanied by metabolic changes, fever or other signs of infection, and lasting for a period of at least 48 hours accompanied by objective change of at least 0.5 in the EDSS score. Confirmed relapses and EDSS scores were recorded consecutively. Time from baseline gene expression analysis to next acute relapse was recorded and used as a variable for clinical outcome prediction.

### RNA isolation and microarray expression profiling

The blood samples were collected for this analysis. After blood withdrawal, PBMC was immediately purified and frozen in liquid nitrogen for the future microarray analysis. Microarray analysis was performed each time that a large enough set of samples was collected (between 10–12 samples for a working set).

PBMC were separated on Ficol-Hypaque gradient, total RNA was purified, labelled, hybridized to a Genechip array

(either HU-133A or HU133A-2) and scanned (Hewlett Packard, GeneArray-TM scanner G2500A) according to the manufacturer's protocol (Affymetrix Inc, Santa Clara, CA, USA). All microarrays used in analysis passed all the stringent quality control criteria. The gene expression measurements used in this study are available and can be downloaded from Gene Expression Omnibus (; accession number GSE15245).

### Data Analysis

Data analysis was performed by Partek Genomics Solution software . Expression values were computed from raw CEL files by applying the Robust Multi-Chip Average (RMA) background correction algorithm. The RMA correction included: 1) values background correction; 2) quantile normalization; 3) log2 transformation; 4) median polish summarization. In order to avoid the noise caused by variable set effects we normalized each set to pre-saved distribution pattern of a well balanced set used as a reference distribution.

To reduce batch effect, Analysis Of Variance (ANOVA) multiple model analysis was applied. Source of variation was analyzed; non-relevant batches effects such as array type, working batch, patient age and gender were eliminated. Most Informative Genes (MIGs) were defined as genes that distinguished between the different classes of time to next acute relapse with p < 0.01 by ANOVA test. Most Correlated Genes (MCGs) were defined as genes that were correlated with time to next acute relapse by Spearman, Pearson or Kendal method with p < 0.05.

### Dividing the patients to classes

We divided the patients to three classes comprising at least 20 patients. Each class corresponded to relatively similar time ranges until their next relapse: a) 31 patients that had not experienced relapse in the 1264 days of the follow up period; b) 40 patients that experienced relapse in less than 500 days and c) 23 patients that experienced relapse in 500 to 1264 days. The boundaries of the classes were chosen according to various constrains: 1) the upper bound (1264) reflects that the patients' follow up period after blood withdrawal. 2) We wanted to divide the time rang 0–1264 to relatively similar ranges such that the number of patients in each group will be similar (at least 20 patients). Thus, we decided on the second boundary (500), a larger boundary would decrease the number of patients in the second group while a smaller one would result in a too small time range of the first group.

The distribution of subjects with CIS and Definite MS across these three categories is [30:16:15] for definite MS and [9: 5:14] for CIS for the three classes (less than 500 days: between 500 and 1264 days: more than 1264 days) respectively. As can be seen, in both groups there are subjects in each of the three categories.

In the case of the FTP, we used resolution of 50 days since we wanted to divide the range related to the first group to equally spaced sub-ranges (thus, for example, 30 or 90 days were inappropriate). It is important to note that the concept used here could be used with different resolutions and still give qualitatively similar results (for example lower resolution of 100 days gave error-rate of less than 0.2, p < 0.0001).

### Implementations of the predictors

The predictor has two major parts: 1) a Support Vector Machine (SVM) classifier, that we named First Level Predictor (FLP), and 2) a multivariate linear regressor that we named Fine Tuning Predictor (FTP). We used the Spider  implementation for the SVM multi-class classifier. We used a multivariate linear regressor for the FTP (for patients with time till next relapse < 500). This multivariate linear predictor gave better results than SVM regressor (the Partek implementation of SVM regressor gave error rate that was much higher, around 0.8).

In the case of the FTP, the gene expression of each predictive gene is multiplied by a weight (positive or negative real number) and the results are summed. This sum is used as a prediction for the number of days till the next relapse. Thus, the expression level of each gene may have a positive or a negative affect on the prediction. In the case of the FLP, which is based on SVM classifier [31], the general idea is similar but more complicated – each gene may have a positive effect on the prediction in some cases and negative effect in other cases.

### The feature selection procedure for finding the most predictive genes

We used different sets of genes as input to our feature selection procedure: a) genes that were differentially expressed between the three different classes of time till the next relapse, b) genes that were expressed with correlation (p < 0.05) with the time till the next relapse, c) all unique genes presented on the microarray.

Using a very large set of genes for constructing the predictors elevates the risk of overfitting (*e.g. *see [32]). The solution for overfitting problem is the Leave 20% Out Cross validation (L20OCV) procedure (a version of leave one out cross validation [33,34]) that is described in this subsection.

**Figure 1:**
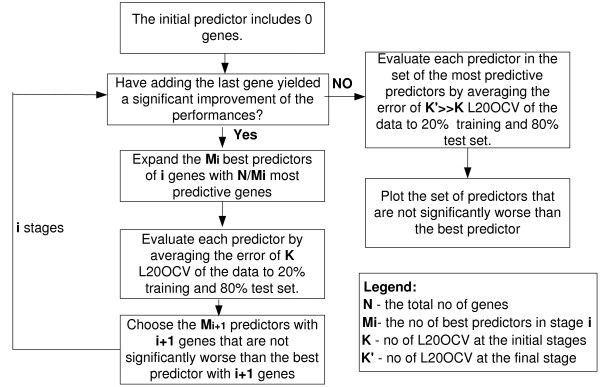
**General flow diagram of the procedure for finding the predictors (FLPs or the FTPs, we used *k *= 100 and *k' *= 10,000)**.

A general flow diagram of the forward selection algorithm for choosing the set of genes consisting each of the predictors is described in Figure 1. The same feature selection algorithm was used both for the FLP and for the FTP.

We started with a set of 22,215 gene-transcripts present on each microarray. The expression levels of multiple probes related to the same gene were averaged, resulting in a set of 10,594 potential features (genes and annotated sequences). At the initial stage, we computed a vector of errors in 100 (80% training set, 20% test set) leave 20% out iterations for each gene. We chose the gene with the lowest mean error rate and all the genes whose mean error rate that is not significantly higher than the error rate of the first gene (p-value < 0.05 by t-test).

In the following stages, we expanded the initial sets while getting significantly better predictors which were based on 2, 3 or more genes. In each step, we tried to expand each predictor in the current set of best predictors by adding more genes to the predictors, and while keeping all the predictors whose error rates were not significantly worse than the error rate of the best predictor. This was done by an iterative cross validation procedure in which 20% of the initial data set was left apart and the remaining 80% were used as temporary training set in each of the iterations. We performed 100 iterations for the cross validation procedure at each stage for selecting sets of potential predictors. The output of the initial stage was a set of predictors with similar performances [*i.e. *according to the 100 leave 20% outs, none of them was significantly better than the best predictor; namely, all the p-values were > 0.05].

At the final stage, to discriminate between the predictors that have similar performances as the best predictor, we performed 10,000 cross validation iterations and selected the predictor(s) that was/were significantly better than the others.

Note that qualitatively similar results were gained when we learned the predictors based on 85 of the patients and afterward test them on a different group of 9 patients (the rest of the patients). In this case, the mean error rate of the FLPs was less than 0.11 on the training set and 0.13 on the test set (all p-values < 0.001). In the case of the FTPs, the mean error rates on the training set and test set were 0.44 and 0.66 respectively (all p-values < 0.001).

### Evaluating the Performance of the Predictors on Subgroups of Patients

For evaluating the performances of the predictors on subgroups of the patients (*e.g. *MS *vs. *CIS, or non-treated *vs. *patients under the various treatments), we performed 1000 Leave 20% Out Cross Validation (L20OCV) procedures where in each L20OCV step we randomly chose subsets of 80% of *each *of the patient subgroups for training the predictors (all these subsets were unified to one subset), and tested the predictors on the rest of the samples (in the cases where the dataset was very small, at least one patient was chosen for the training set and for the test set). The predictors were based on the sets of genes that were found by the procedure that was described in the previous subsection, but in each iteration a different training set for inferring the weights of the different genes in the predictor was used; and in each iteration the predictors were implemented on different test set. The final error rate of each subgroup is the average error rate across all the 1000 L20OCV procedures for patients from the subgroup.

### Another Set of Patients for Additional Validation

For further evaluation of the FLP performances, we collected an additional dataset of 10 patients (3 from the first group, 2 from the second group, and 5 from the third group). The gene expression of each of the patients was normalized separately with the original dataset of 94 patients. Then, the FLPs were implemented on the normalized expression levels. The demographical and clinical characteristics of these patients are presented in Table 3.

**Table 3:** Clinical and demographical characteristic of the additional validation set of patients (4 CIS and 6 Definite MS).

	**Age (Years)**	**Disease Duration (Years)**	**Annua Relapse Rate**	**EDSS**	**F/M**	**Yes/No Future IMD Treatment**
**CIS**	24 ± 5.01	0.34 ± 0.09	6.1 ± 2.05	2.58 ± 0.15	0/6	6/0

**Definite MS**	36 ± 7.61	5.3 ± 2.39	1 ± 0.51	5.3 ± 2.39	2/2	3/1

### The Role of Clinical and Demographical Variables in the Predictors' Performance

We examined if clinical parameters are helpful for predicting the time to next relapse by 1) evaluating the performance of a FLP and FTP predictors that are based only on these parameters, and 2) examining if these variables can improve the performances of our predictors. To this end, we checked the following clinical parameters: age, MS stage (CIS or Definite), gender, annual relapse rate, EDSS at time of blood sampling, disease duration, age at onset, EDSS change in the last relapse.

### Empirical p-values for the predictors

We computed empirically p-values for the best FLP and FTP by performing random permutation of the labels, learning best predictors for each such permutation, and computing the fraction of cases (out of 1000 permutations) that a predictor for permutated labels gave better error rate than the original predictor. All the 1000 random predictors were much worse than the original one (p-value < 0.001). For example, the average error rates of the random FLP were 0.67 and the average error rates of the random FTP were 0.8.

### The Role of Relapse Severity in the Predictors' Performance

We examined if the performances of the predictors depend on the severity of the relapse (measured as the change in EDSS in the last relapse). To this end, we divided the patients to 8 groups according to their change in EDSS in the last relapse (the range was 0.5 – 6.5). We computed the error rate of the predictors for each of these groups (as was performed for the treatment groups).

### Biological functional analysis

Gene functional annotation was performed using functional classification tools such as David Bioinformatics Resources  and Ingenuity Pathways Analysis web-software . Information about the most predictive genes was extracted from NCBI .

## Results

To learn about the three groups of patients, we performed Principal Component Analysis (PCA) and clustering analysis of the patients based on 1359 MIGs (Additional file 2). The patients with time until next relapse < 500 days exhibit a relatively coherent clustering where 29/40 of the patients appear in the same cluster. The clustering results of the other groups were much worse as they were partitioned among many clusters with up to 5 patients in the same cluster. This result demonstrates that it is much more complicated to cluster sub-groups of patients that will experience their next relapse in more than 500 days. Thus, an additional, finer predictor only for the first group (time until next relapse < 500) was justified.

We named the three groups classifier First Level Predictor (FLP). The FLP classified the time till next relapse of a patient to one of the following groups: <500 days, between 500 and 1264 days, and >1264 days. The finer predictor, for the patients with time till next relapse < 500 days was named Fine Tuning Predictor (FTP).

**Figure 2:**
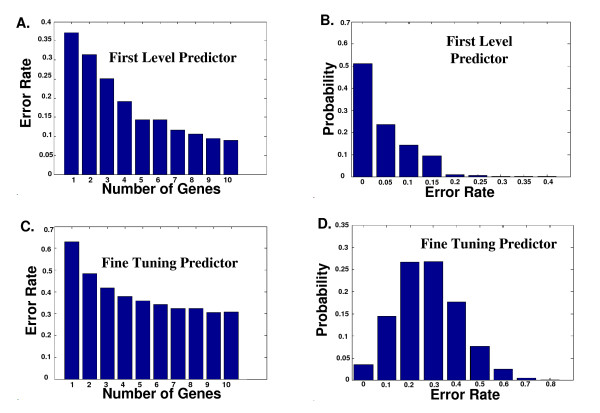
**Error probabilities of the FLP and the FTP**. A. The improvement in the error probability of the best FLP as function of the number of predicting genes. Each improvement was significantly better (p-value < 0.05) than the previous one (see Methods). B. The error rate distribution of the best FLP on the test sets. C. The improvement in the error probability of the best FTP. Each improvement was significantly better (p-value < 0.05) than the previous one (see Methods). D. The error rate distribution of the best FTP on the test sets.

Based on expression of all 10,594 genes, we performed a Leave 20% Out Cross Validation (L20OCV) procedure to evaluate FLP that simultaneously discriminated between patients that experienced acute relapse in one of the three periods mentioned above during clinical follow up (as was described in the Methods section). The cases where the FLP does not classify patients to their correct groups are defined as errors. The output L20OCV was a group of predictors that are based on the gene expression of sets of 1 to 10 genes with error rate range between 0.37 (for single gene) to 0.079 (for 10 genes), see Figure 2A. There are a few dozen predictors that gave similar results. For example, Additional file 3 includes 36 FLP with error rate < 0.1.

To differentiate a smaller set of statistically significant predictors from those with similar predictive ability we performed 10000 additional L20OCV iterations which enabled us to choose three best FLP (each is based on 10 genes; see Table 4).

**Table 4:** The performances (error rate) of the predictors in different subgroups of patients.

**Predictor**	**CIS**	**Definite MS**	**Non treated**	**Interferon beta 1a Avonex**	**Interferon beta 1b Betaferon**	**Interferon beta 1a Rebif**	**Glatiramer acetate Copaxone**	**Intravenous Immunoglobulines Iv-Ig**	**Average error rate**
**FLP1**	0.10	0.08	0.087	0.01	0.01	0.09	0.18	0.175	0.079
**FLP2**	0.10	0.08	0.085	0.02	0.03	0.05	0.215	0.18	0.0791

**FLP3**	0.088	0.071	0.082	0.01	0.02	0.105	0.165	0.17	0.0792

**FTP1**	0.21	0.355	0.332	--	--	0.42	0.54	0.34	0.345

**FTP2**	0.29	0.38	0.366	--	--	0.345	0.51	0.3	0.349

**3FTP**	0.27	0.37	0.366	--	--	0.245	0.535	0.41	0.349

**FTP4**	0.31	0.38	0.352	--	--	0.235	0.53	0.37	0.349

FLP1 – FLP3 denote FLPs and FTP1 – FTP4 denote FTPs; see Table 5 for the genes in each predictor) on each sub-group (when applicable), and the average error-rate (last column). The average error rate is the error rate when considering the entire dataset.

The improvement in the error rate of the FLP as a function of the number of genes that were used by the predictor is demonstrated in Figure 2A. Genes were added iteratively to the FLP until there was no significant improvement in the performances of the predictor (a flow diagram appears in Figure 1).

The error rate distribution of the best FLP is depicted in Figure 2B. As can be seen, for 50% of patients the predictor gave absolutely correct classifications (*i.e. *it correctly classified all the patients to one of three groups), in 20% of the cases the error rate was 0.05, and only 30% of patients were predicted with error rate more than 0.05. The 10 genes involved in each of the 3 best FLPs are presented in Table 5. The functional annotations of those genes appear in Additional file 4.

**Table 5:** The genes that were selected for the best FLPs (FLP1 – FLP3) and best FTPs (FTP1 – FTP4).

Predictor	Predictor name	Error Rate	Gene 1	Gene 2	Gene 3	Gene 4	Gene 5	Gene 6	Gene 7	Gene 8	Gene 9	Gene 10
FLP	FLP1	0.079	FLJ10201	PDCD2	IL24	MEFV	CA2	SLM1	CLCN4	SMARCA1	TRIM22	TGFB2
	
	FLP2	0.0791	FLJ10201	PDCD2	IL24	MEFV	CA2	SLM1	CLCN4	SMARCA1	TRIM22	SPN
	
	FLP3	0.0792	FLJ10201	PDCD2	IL24	MEFV	CA2	SLM1	CLCN4	SMARCA1	TRIM22	TP73L

FTP	FTP1	0.345	KIAA1043	LOC51145	PPFIA1	MGC8685	DNCH2	PCOLCE2	FPRL1	G3BP	RHBG	---
	
	FTP2	0.349	KIAA1043	LOC51145	PPFIA1	MGC8685	DNCH2	TAF4B	FPRL1	PCOLCE2	FLJ21802	
	
	FTP3	0.349	KIAA1043	LOC51145	PPFIA1	MGC8685	DNCH2	PCOLCE2	FPRL1	FLJ21802	TAF4B	
	
	FTP4	0.349	KIAA1043	LOC51145	PPFIA1	MGC8685	DNCH2	PCOLCE2	FPRL1	TAF4B	FLJ21802	

Full description of the genes and their gene bank ID appears in Additional file 4. The expression levels of the genes appear in Additional file 1. MEFV is an abbreviation of Homo sapiens familial Mediterranean fever locus region, mRNA sequence.

**Figure 3:**
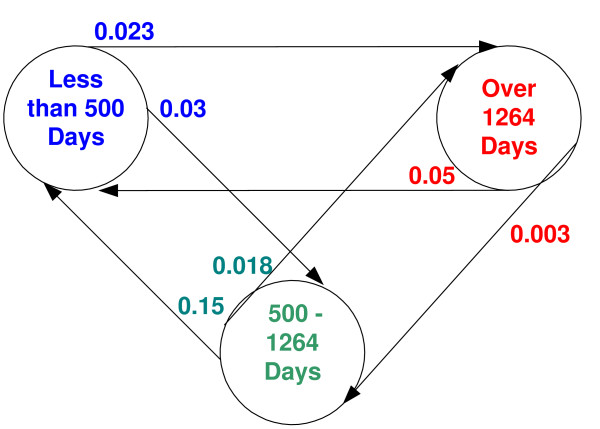
**The different types of errors of the best FLP (FLP1)**. The number on an arrow from state *x *to state *y *is the probability that the predictor miss-classify a patient whose true state is *x *and will put it in state *y*. This is an extension of the widely used two states sensitivity and specificity measures.

The probabilities of the different types of classification errors of the best FLP are depicted in Figure 3. For example, as can be seen in Figure 3, a patient that belongs to group 1 (relapse in less than 500 days) has a probability of 0.03 of being misclassified by the FLP and to be included in group 2 (relapse in 500 – 1264 days), and a probability of 0.023 to be misclassified and to be included in group 3 (relapse in more than 1264 days). In comparison, a random assignment to one of these groups gave an error rate of 0.67.

The performances of the predictor were significant (p < 0.001; details about the p-value calculation appear in the Methods section).

When we implemented the feature selection procedure using only MIG genes the performances of the result FLP were not improved (see Supplementary Note 1 in Additional file 5).

**Figure 4:**
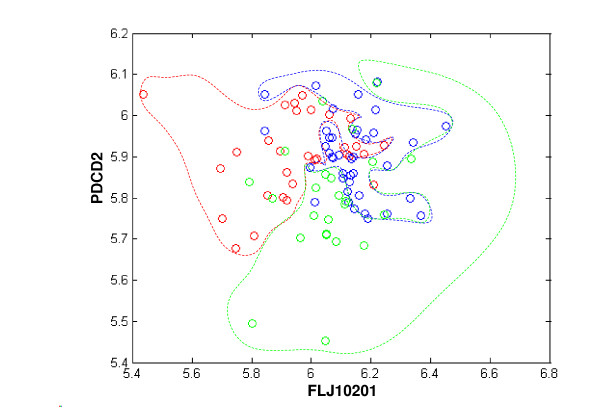
**The three classification regions (relapse in 500 days – blue, relapse in 500–1264 days – green, relapse in more than 1264 days – red) as function of the gene expression of the two most predictive genes of the FLP, *FLJ10201 *and *PDCD2***. Each point denotes a patient.

To visualize boundaries of the FLP decision regions we performed a plot of the expression levels of the 2 most predictive genes (*FLJ10201 *and *PDCD2*) of the best FLP and the boundaries of the decision regions of the predictor (Figure 4). As can be seen, the boundaries of these regions are non-linear (see Additional file 6 for graphs of other pairs of predictive genes), and resemble the results that were reported in [26]. Finally, the error rate of the best FLPs on an additional independent dataset of 12 patients was 0.3 (p-value < 0.001; see Methods); further supporting the viability of the FLPs.

Next, we designed a more accurate predictor that was named Fine Tuning Predictor (FTP). It predicts the time until the next relapse only for patients that experience acute relapse during a period of 500 days. As a FTP we used a multivariate regressor (see Methods) that can predict the time until the next relapse with a resolution of a few days. In the case of the FTP, we defined a prediction error as a prediction that is more/less than 50 days (± 50) from the real date of relapse onset. We found 240 gene sets that gave error rate < 0.36. Our feature selection procedure combined with 10000 permutations of Leave One 20% Cross Validation (L20OCV) procedure found four FTP s; each FTP was based on 9 genes. The minimal error-rate of each FTP was 0.35 (p-value < 0.001); and was significantly better than the other gene sets. The error rate of the FTP after random permutations of the labels was 0.8; this is 2.3 folds higher than the error rate of the inferred FTP (see Methods for description about the p-value). The error rates of best 9-genes-FTPs are demonstrated in Table 4.

As in the case of the FLP, we performed a similar analysis of the improvement in the error rate of the best FTP (see Table 4) as function of the number of predictive genes (from 1 to 9; Figure 2C). Every time a gene was added to the FTP, the performances of the FTP were significantly improved (see Methods).

**Figure 5:**
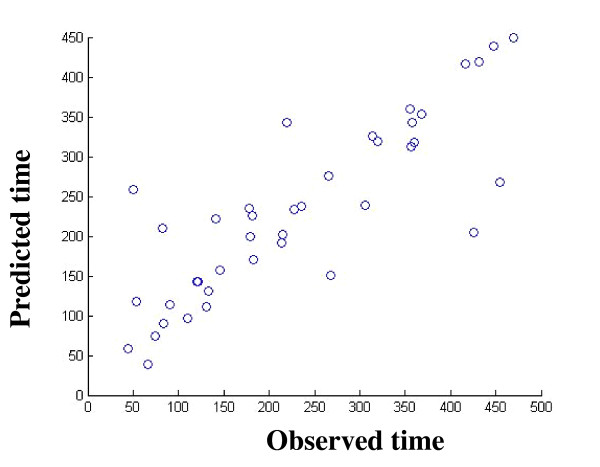
**The predicted time to next relapse versus observed time of the next relapse (in days) for the best FTP**. The graph demonstrates a very high correlation between these two values (Spearman correlation 0.82, p-value = 10^-10^).

The plot of best FTP performances *vs. *observed time to next relapse during 500 days of follow up appears in Figure 5. As can be seen, the two values are very correlative (Spearman correlation 0.82, p-value = 10^-10^). The analysis of error rate distribution of the best FTP appears in Figure 2D. In this case, the error rate has normal distribution with mean error rate of 0.35; for example, 20% of the patients have error rate < 0.2 (Figure 2D).

When we implemented the feature selection procedure only on genes whose expression is correlative with time until next relapse the FTP results were not improved (see Supplementary Note 2 in Additional file 5).

To further evaluate the performances of the predictors, we divided the patients in two ways. First, the dataset was divided to CIS and definite MS patients (32 and 62 patients respectively); second, the dataset was divided into 6 groups according to their *future *treatment after their blood was withdrawn (Non-treated, *Interferon β-1a (Avonex*), Interferon *β*-1b (Betaferon), *Interferon β-1a *(*Rebif*), Glatiramer Acetate (Copaxone) and *Intravenous Immunoglobulins (Iv-Ig*) with 61, 5, 2, 10, 10, and 6 patients respectively (see Table 2). The performances of the best predictors in different subgroups of patients were evaluated as was described in the Methods section.

Table 4 depicts the performances of each of the best predictors (FLPs and FTPs) on each of these groups. The error rates remain significant for each of these groups. The FLP error rates were 0.1 and 0.08 (p-value < 0.001) for the CIS and MS groups respectively; the FTP error rates were 0.21 and 0.355 (p value < 0.001), for the CIS and MS groups respectively.

In the analysis of the different treatments, the *FLP *error rate ranged between 0.087 (non-treated) to 0.18 (*Copaxone*); the *FTP *error rate ranged between 0.235 (*Rebif*) and 0.53 (*Copaxone*), see Table 4. The probabilities of the different types of classification errors for each treatment group appear in Additional file 7. In all sub-groups the best predictor exhibited significant performances (p-value < 0.001); this fact suggests that the signal of the next relapse is usually strong enough to be detected by our predictor even when the patients undergo various Immunomodulatory Drugs (IMD) treatments after blood withdrawal.

For additional validation of the FLP, we collected an additional independent dataset of 10 untreated RRMS patients (see more details about this dataset in the Methods section and Table 3). The total error rate of the FLP on these patients was 0.25. This result further supports the viability of our approach.

Clinical and demographical confounders did not improve the performance of the best gene expression based FLP and FTP (see Methods for the exact list of Clinical and demographical confounders we checked). FLP and FTP predictors that were based on combination of these confounders and that were found by our approach had much higher error rates than the predictors that were based only on gene expression (FLP error rate > 0.63, FTP error rate > 0.74; for all the predictors that were based on the above mentioned confounders). We also did not get a significant correlation between the error rate of a predictor and the severity of relapse (as was measured by the change in EDSS), Spearman correlation -0.29, p-value = 0.5, for FTP; Spearman correlation -0.6, p-value = 0.12, for FLP, see Methods for more details).

## Discussion

In this work, we demonstrated that gene expression in PBMC can be used for predicting the time of the upcoming relapse in MS. Using different prediction strategies to determine an appropriated gene set for accurate relapse prediction we found that the classifier that was based on all microarray genes had the best prediction. We describe a FLP, which is based on the expression levels of ten genes, which can predict the time till next relapse in a resolution of 500 days during 3.5 years of disease progression. An additional FTP, which is based on different set of nine genes, can be used for a prediction of a higher resolution (*e.g. *a resolution of 50 days).

At first glance, the error rate (about 0.35) of the FTP seems relatively high. However it is important to remember that the definition of error in this case was very tight (more than 50 days from the real value). As mentioned, this error rate was very significant (p-value < 0.001) and surprisingly good. For comparison, our simulation showed that a random FTP (*i.e. *the best FTP after a random shuffling the input labels) gave an error rate that was close to 0.8 – 2.3 fold higher than our error rate.

This study includes 94 patients for evaluating the predictors. It is clear that a larger dataset will give better performances. In order to estimate the potential improvement in the error rate when using larger datasets we performed the following analysis: we computed the error rate of the FLP and the FTP as function of the dataset size (% of the original dataset; see Additional file 8). The figure shows that the error rates decrease for larger datasets. This fact suggests that with the accumulation of more gene expression measurements we can design better predictors. Specifically, enlargement of the dataset to 200 patients (instead of 94) will give a classification error of about 0.05 and a regression error of about 0.2 (Additional file 8).

Another interesting conclusion from this work is that there are multiple predictors (FLPs and FTPs) that have similar performances. The predictors that were described in this work were significantly better than the other predictors; however, there were a few dozen predictors that gave similar results. For example, Additional file 3 includes 36 FLP with error rate < 0.1, and 240 FTP with error rate < 0.36. This means that the best predictors appear in this work can be replaced by other predictors with a relatively small influence on the error rate.

Finding a good predictor for the time to next relapse and finding a molecular explanation for relapses are different tasks with a possible overlap. There are a few explanations why the connection between the predictive genes and relapse associated mechanism is not necessarily immediate: First, the predictors were designed to include relatively small number of genes while the actual mechanisms may include dozens of signaling pathways. Second, our study was based on changes in gene transcription levels; it is possible that major parts of the relapse associated regulatory mechanisms are post transcriptional. In such cases, the most relevant genes are useless in terms of improving the predictions and the feature selection procedure finds genes that are less relevant but that exhibit significant change in their mRNA levels (e.g. genes that are regulated/regulate the genes that are directly related to relapse).

However, many of the FLP genes are linked to MS. This is an additional support of their predictive ability. For example, the gene *TGFB2 *is a one of the master genes in MS; it is closely related to a rapid recovery from relapses that is mediated by *Th2/Th3 *lymphocytes. *Th2/Th3 *lymphocytes produce anti-inflammatory cytokines (like *IL10*) [35]. *TGFB *inhibits *IL12 *mediated inflammatory response, and it virtually decreases T cells proliferation and *IFNgamma *production [36]. *TGFB *prevents induction of pro-inflammatory gene-program by inhibiting the expression of 25% of the *TNFalpha/IFNgamma *induced genes [35]. The target genes that *TGFB *inhibits are various genes that are involved in MS pathogenesis processes (*e.g. *chemotaxis, adhesion and cell migration).

The gene for Familial Mediterranean Fever (*MEFV*) is expressed in early leukocyte development and is regulated in response to inflammatory mediators. Stimulation of cells with the proinflammatory agents interferon (*IFN*) gamma, tumor necrosis factor, and lipopolysaccharide induced *MEFV *expression, whereas the anti inflammatory cytokines (*IL4, IL-10*) and especially *TFGB *inhibited such expression [37].

The *CA2 *gene (carbonic anhydrase II) supports the transport of bicarbonate ions, sodium ions, and water from blood to the *CSF*; and in the myelin sheath *CA2 *supports compaction of myelin by stimulating co-transport of ions between the myelin membranes. The double mutant mice deficient by *CA2 *and myelin displayed tremors and seizures [38]. Interestingly, the onset of seizures was delayed significantly in the double mutants, and the lifespan increased by several months, this fact corroborates with CA2's activity as predictor of acute onset in *MS*.

Another important group of genes that are part of the best FLP is related to the interferon regulation mechanism. This group includes RNA binding and signal transduction *SLM1 *gene, that is associated with *Interferon Receptor 1 Binding Protein 4 *(*IR1B4*) [39] and *TRIM22*, an important member of interferon related genes, that is involved in transduction of *IFN *activity [40].

**Figure 6:**
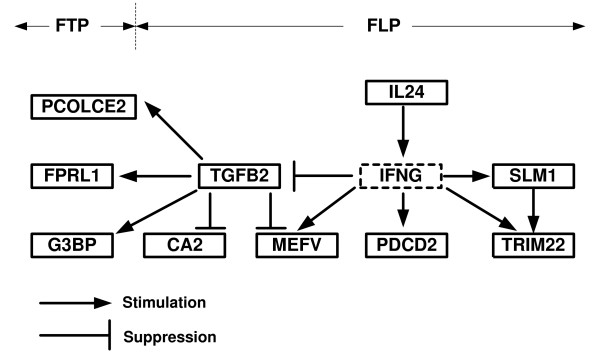
**Regulatory network of the genes that are part of the best FLP and FTP; the network is based on the literature (see the Discussion section)**.

The IL24 gene is a member of anti-inflammatory *IL10 *family cytokines involved in immune response. The over-expression of *IL24 *stimulates pro-apoptotic *CADD *family genes and activation of apoptosis. On the other hand, IL24 can increase secretion of IFNG in human PBMC. The IFNG by himself is able to repress TGFB mRNA expression as demonstrated in CD18 positive cells [41] and in human lymphocytes it increases mRNA expression of MEFV [42]. Additionally IFNG involved in regulation of the protein NFKBIB (corresponding to the gene PDCD2) that is associated with programmed death of lymphocytes [43-45]. Based on the above relations we reconstructed a unified regulatory network for most of the predictive genes that appear in the FLP (see Figure 6).

The best FTP includes 2 inflammatory related genes (FPRL1 and PPFIA1). FPRL1 functions as a receptor component of inflammatory response [45], activation of FPRL1 results in leukocytes activation. FPRL1 involved in direct monocytes/microglia migration as was demonstrated in activated brain prion plaques and brain lesions in Alzheimer disease [46]. PPFIA1 receptor gene is involved in cell motility, cells spreading, migration and adhesion. Up regulation of human PPFIA1 (LIPRIN) gene in peripheral blood is associated with psoriatic arthritis [47]. Another interesting gene is G3BP that encodes a downstream effector protein of the Ras signaling pathway [48]. Interestingly, as in the case of best FLP, the genes FPRL1 and G3BP are regulated by TGFB [49,50]. This result suggests that a single pathway can explain both the FLP and the FTP genes.

To summarize, most of the predictive genes seem very relevant to the pathophysiology of MS. We have constructed a schematic regulatory network which unifies many of these genes to a single regulatory network (Figure 6). One important goal for further research is to better understand how these genes are involved in the biological mechanisms that lead to a clinical relapse.

We also found a few predictive genes whose their biological roles are unknown (see Additional file 4). We thus think that the potential connection of these genes to MS is a natural target for a further study.

As relapses have different level of severity [e.g. it can be measured by the increase in EDSS] one may think that in the cases of a more severe next relapse the performances of the predictors will be better. Analysis of the predictor error probability for the FLP and FTP as a function of the change in EDSS levels showed a negative relation (as expected – *i.e. *larger changes in EDSS are easier to detect). However, this correlation was not significant. Thus, a final answer to this question should be deferred till a larger gene expression dataset will be accumulated.

We used a heterogeneous dataset for inferring the predictors. The dataset included both CIS and Definite MS patients, and patients that underwent different immunomodulatory treatments after their blood was sampled. A survival analysis showed that the disease stage (CIS or Definite MS) had a statistical significant influence on the probability to experience next acute relapse (Additional file 9, Supplementary Note 3 in Additional file 5). Further, statistical analysis of the gene expression of these two groups of patients showed that there are dozens of genes that are differentially expressed in these groups (data not shown).

However, our predictor was insensitive to the disease stage and successfully dealt with this issue. The error rates for the two groups were significantly low (less than 0.1 for the FLP and less than 0.37 for the FTP). This fact may suggest that, in the case of the small sets of predictive genes, the changes in PBMC gene expression before the second relapse (CIS patients) or before any other relapse (Definite MS patients) are similar.

Our dataset includes patients that underwent various treatments after we sampled their gene expression. We believe that these patients are the major source of error for our predictors. On the other hand, we decided to include them in the analysis since they improved its statistical significance. We demonstrated that our predictor gave significantly good results, also when considering each of these datasets separately. This was unexpected since it is known that in general drugs change the relapse frequency. The explanation of this result is simple: Most of the treatments delay the next relapse by about 30% [51-55], and this fact increases the prediction error primarily for patients whose real time to next relapse is close to the boundaries of their classification group (*e.g. *close to 500 or close to 1264). Since the number of such patients is relatively low the error rate remains significantly low (see details in Supplementary Note 4 in Additional file 5).

## Conclusion

We conclude that gene expression in PBMC can be used to accurately predict the time until the next acute relapse. In this work, we described a few sets of predictive genes that can be used for this purpose and demonstrated that other combinations may also yield significant results. It is possible that different technology for measuring the gene expression will yield different sets of the most predictive genes. Thus, our next goal is to find sets of predictive genes that give significant results when their gene expression is measured by cheaper, small-scale, technologies such as kinetic RT-PCR.

In this work, as information about clinically silent lesions was not available for most of the patients, we focused only on clinically definite MS relapses. In the future, when such information will be available, it can be used for improving the performances of our predictor. In addition, based on such data it will be feasible to study the possibility to predict radiological MRI lesions (that are possibly clinically silent) from gene expression in PBMC.

Finally, it is possible that the techniques described here will be valuable not only in future MS research but also in other autoimmune disease with relapsing-remitting nature.

## Abbreviations

CIS: Clinically Isolated Syndrome; PBMC: Peripheral Blood Mononuclear Cells; MS: Multiple Sclerosis; MIG: Most Informative Genes; MCG: Most Correlated Genes; SVM: Support Vector Machine; L20OCV: Leave 20% Out Cross Validation; FLP: First Level Predictor; FTP: Fine Tuning Predictor; RRMS: Relapsing Remitting *MS*; EDSS: Expanded Disability Status Scale; RMA: Robust Multi-chip Analysis; ANOVA: Analysis of Variance; IMD: immunomodulatory drugs.

## Competing interests

The authors declare that they have no competing interests.

## Authors' contributions

MG, TT, and AA participated in the design of the study. TT and UR designed the predictors. MG, TT, and RO analyzed the data. MG, TT, and AA wrote the paper. All the authors approved the final manuscript.

## Pre-publication history

The pre-publication history for this paper can be accessed here:



## Supplementary Material

Additional file 1 **Supplementary Table 1**. The previous treatment of each patient and the number of days between the previous treatment and blood sampling; the expression levels of the predictive genes across all analyzed patients and the number of days till the next relapse for each of the patients. Click here for file

Additional file 2 **Supplementary Figure 1**. Clustering and Principal Component Analysis (PCA) analysis of the patients based on gene expression of 1359 MIGs. Click here for file

Additional file 3 **Supplementary Table 2**. FLPs/FTPs whose performances are similar to the performances of the best FLP/FTP described in the main text. Click here for file

Additional file 4 **Supplementary Table 3**. Description of the predictive genes. Click here for file

Additional file 5 **Supplementary notes 1–4**. All the Supplementary notes. Click here for file

Additional file 6 **Supplementary Figure 2**. The region of each of the classes as function of the gene expression of the predictive genes. Click here for file

Additional file 7 **Supplementary Figure 3**. The different types of errors of the best FLP (FLP1) for different stages of the disease and for different future IMD treatment. Click here for file

Additional file 8 **Supplementary Figure 4**. The prediction errors as function of the dataset size (% of the used dataset) for the FLP and the FTP. Click here for file

Additional file 9 **Supplementary Figure 5**. Survival analysis of MS patients with definite MS and patients with Clinically Isolated Syndrome. Click here for file
