# Supplementary material for: Prediction of acute multiple sclerosis relapses by transcription levels of peripheral blood cells
Source: BMC Med Genomics. 2009 Jul 22;2:46. doi: 10.1186/1755-8794-2-46 (PMC2725113; doi:10.1186/1755-8794-2-46)
Supplement: Additional file 2 — Supplementary Figure 1. Clustering and Principal Component Analysis (PCA) analysis of the patients based on gene expression of 1359 MIGs. [file 1755-8794-2-46-S2.doc]

**Supplementary Figure 1: Clustering (A.) and Principal Component Analysis (PCA) analysis (B.) of the patients based on gene expression of 1359 MIGs. Red color denote patients that will experience relapse in <500 days, blue denote patients that will experience relapse in 500-1264 days, green denote patients that will experience relapse in > 1264 days. The figure demonstrates that when considering MIGs it is much easier to cluster the patients that will experience relapse in <500 than the patients in the two other groups.**
