# Supplementary material for: Prediction of acute multiple sclerosis relapses by transcription levels of peripheral blood cells
Source: BMC Med Genomics. 2009 Jul 22;2:46. doi: 10.1186/1755-8794-2-46 (PMC2725113; doi:10.1186/1755-8794-2-46)
Supplement: Additional file 5 — Supplementary notes 1–4. All the Supplementary notes. [file 1755-8794-2-46-S5.doc]

# Supplementary Notes (Notes 1-5) for the paper: “Prediction of acute multiple sclerosis relapse by transcriptions levels of peripheral blood cells”

*Michael Gurevich*, Tamir Tuller*, Udi Rubinstein, Rotem Or-Bach and*

*Anat Achiron*

**Supplementary Note 1: Using only the MIG genes does not imply a FLP with better performances.**

We compared the prediction error of the best FLP (which is based on all genes on the microarray platform) to a classifier that used only genes that were significantly distinguished between the different classes of time to next acute relapse (MIGs; Methods). When our feature selection algorithm was applied on the set of 1359 MIGs the error rate increased by 77% (error rate of 0.14 instead of 0.079, for a best FLP that is based on all the genes).

**Supplementary Note 2: Using genes whose expression is correlative with time until next relapse does not imply a FTP with better performances.**

We checked if implementing the feature selection procedure on a set of genes whose expression was correlated with the time to next relapse can improve the performances of the FTP. For this purpose, we identified a set of 1278 genes whose expression is significantly (p<0.05) correlated with time to next relapse (we consider at least one of the three types of correlations: Spearman, Pearson, Kendal) as candidate genes for our feature selection procedure. The resulted FTP gave an error rate that is 31% worse (0.46 instead of 0.35, p-value < 10^-16^) than the original one (*i.e.* when we used all the genes).

**Supplementary Note 3: Different immunomodulatory treatments for patients with CIS or with Definite MS can not explain significant difference in the probability to experience next acute relapse between these two groups**

The probability to experience next acute relapse is higher for patients with Definite MS. All the CIS patients were untreated in the period after their blood was withdrawn. Thus, the fact that some of the Definite MS patients were treated should only decreased the gap between these two groups (without treatment, these Definite MS patient would have experienced relapse with even higher probability).

**Supplementary Note 4: Why the performances of the predictors remain significant although some of the patients were treated after blood sampling.**

Most of the treatments delay the next relapse by about 30%.

Our dataset includes 94 patents, 61 (the majority) are not treated and therefore they do not cause any bias. The 33 patients who have been treated after blood sampling are (close to) equally distributed among the three groups.

The patients from the third group (relapse in more than 1264 days) do not bias the predictions. In this case, adding a delay of 30% to the time till the next relapse does not change the fact that the relapse will be in more than 1264 days (*i.e.* their real classification remains the "third group").

In the case of the first group (less than 500 days), the problem is only relevant for patients who experienced their next relapse in more than about 0.77*500 day after blood sampling. In these cases, the treatments have moved these patients to the second group (500-1264 days; i.e. 0.77*500 + 0.3*0.77*500 > 500) and thus the prediction should be incorrect (*i.e.* they will classify as part of the "first group" while their real classification due to treatment is the "second group"). There are about 0.23*10 = 2.3 such patients (assuming uniform distribution of relapse rates in the three groups).

In the case of the second group, the situation is similar to the first group -- the problem is relevant only to the patients that experienced their next relapse in more then 0.77*1264 days (since the treatment will move them to the third group, more than 1264 days, and there will be an error). There are about 0.23*10 = 2.3 such patients (assuming uniform distribution of relapse rates in the three groups).

Thus the total error rate (assuming a perfect predictor) will be less than 5/94 = 0.053 (very close to the error rate that we got: between 0.08 and 0.1).
