# Supplementary material for: Prediction of acute multiple sclerosis relapses by transcription levels of peripheral blood cells
Source: BMC Med Genomics. 2009 Jul 22;2:46. doi: 10.1186/1755-8794-2-46 (PMC2725113; doi:10.1186/1755-8794-2-46)
Supplement: Additional file 6 — Supplementary Figure 2. The region of each of the classes as function of the gene expression of the predictive genes. [file 1755-8794-2-46-S6.doc]

**A.**

**B.** **Supplementary Figure 2. The three most predictive genes of the FLP are *FLJ10201*, *IL24*, and *PDCD2*. The region of each of the classes (relapse in 500 days – blue, relapse in 500-1264 days – green, relapse in more than 1264 days - red) as function of the gene expression of these predictive genes appears with the corresponding color. Supplementary Figure 2A describes these regions in the (*FLJ10201*, *IL24*) plane. Supplementary Figure 2B describes these regions in the (*PDCD2*, *IL24*) plane.**
