# Supplementary material for: Prediction of acute multiple sclerosis relapses by transcription levels of peripheral blood cells
Source: BMC Med Genomics. 2009 Jul 22;2:46. doi: 10.1186/1755-8794-2-46 (PMC2725113; doi:10.1186/1755-8794-2-46)
Supplement: Additional file 7 — Supplementary Figure 3. The different types of errors of the best FLP (FLP1) for different stages of the disease and for different future IMD treatment. [file 1755-8794-2-46-S7.doc]

**Supplementary Figure 3. The different types of errors of the best FLP (FLP1) for different stages of the disease (CIS *vs.* Definite; A.) and for different future IMD treatment (Non treated, Interferon beta 1a -- Avonex, Interferon beta 1b --**

**Betaferon, Interferon beta 1a -- Rebif, Copaxone, Intravenous Immunoglobulines Iv-Ig; B.). The number on an arrow from state *x* to state *y* is the probability that the predictor will miss-classify a patient whose true state is *x* and will put it in state *y* instead.**
