# Supplementary material for: Prediction of acute multiple sclerosis relapses by transcription levels of peripheral blood cells
Source: BMC Med Genomics. 2009 Jul 22;2:46. doi: 10.1186/1755-8794-2-46 (PMC2725113; doi:10.1186/1755-8794-2-46)
Supplement: Additional file 8 — Supplementary Figure 4. The prediction errors as function of the dataset size (% of the used dataset) for the FLP and the FTP. [file 1755-8794-2-46-S8.doc]

**A.**

**B.**

**Supplementary Figure 4. The prediction errors as function of the dataset size (% of the used dataset) for the FLP (A.) and FTP (B.).**
