# Supplementary material for: Prediction of acute multiple sclerosis relapses by transcription levels of peripheral blood cells
Source: BMC Med Genomics. 2009 Jul 22;2:46. doi: 10.1186/1755-8794-2-46 (PMC2725113; doi:10.1186/1755-8794-2-46)
Supplement: Additional file 9 — Supplementary Figure 5. Survival analysis of MS patients with definite MS and patients with Clinically Isolated Syndrome. [file 1755-8794-2-46-S9.doc]

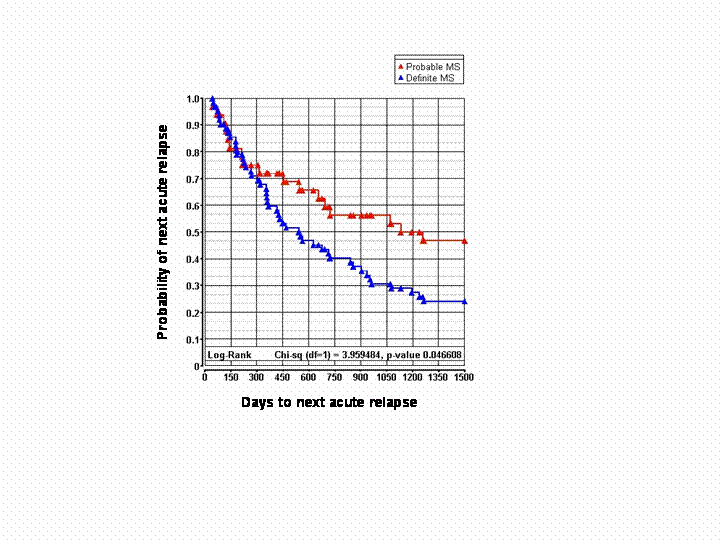
**Supplementary Figure 5: Survival analysis of MS patients with definite MS (blue) and patients with Clinically Isolated Syndrome (CIS, red).** **Each point denotes the probability that the next acute relapse (y-axis) will be in *more* than a certain number of days (x-axis).**
